# Obesity Drug Update: The Lost Decade?

**DOI:** 10.3390/ph3123494

**Published:** 2010-11-24

**Authors:** Fayi Yao, Robert George MacKenzie

**Affiliations:** Department of Psychiatry and Behavioral Neurosciences, Wayne State University School of Medicine Detroit, MI 48201, USA

**Keywords:** obesity, anti-obesity drug targets, anti-obesity drugs, hypothalamus

## Abstract

The growing worldwide obesity epidemic and obesity-related disorders present a huge unmet medical need for safe and effective anti-obesity medications. The discovery of leptin in 1994 was rapidly succeeded by a wave of related discoveries leading to the elaboration of a hypothalamic melanocortinergic neuronal circuit regulated by leptin and other central and peripheral signaling molecules to control energy homeostasis. The identification of specific neuronal subtypes along with their unique connections and expression products generated a rich target menu for anti-obesity drug discovery programs. Over the course of the last decade, several new chemical entities aimed at these targets have reached various stages or successfully completed the drug discovery/regulatory process only to be dropped or taken off the market. There are now in fact fewer options for anti-obesity drug therapies in late 2010 than were available in 2000. The challenge to discover safe and effective anti-obesity drugs is alive and well.

## 1. Introduction

Obesity is a major health problem that results when energy intake exceeds energy output with the excess being stored as fat in adipose tissue and ectopically in other tissues. Developing in an environment of nutritional scarcity, humans evolved to ingest available nutrients and to store and process them efficiently. Consequently, in modern societies marked by nutrient excess with ubiquitous food of high palatability and caloric density and life styles requiring low energy output, obesity has become a growing affliction such that there are now more than 10^9^ overweight people (body mass index, BMI = weight in kg/height in m^2^ > 25) and 300 million of these are obese (BMI > 30) [1].

Excess fat storage is typically accompanied by a low-grade inflammation which, in conjunction with lipotoxicity, leads to insulin resistance of insulin-sensitive tissues such as muscle and liver [2,3,4]. Obesity is therefore a major risk factor for Type 2 diabetes along with other serious pathologies such as dyslipidemias, cardiovascular disease, certain cancers, sleep apnea and stroke. The good news is that obesity can be treated by diet and exercise but the bad news shows that these therapies have poor patient compliance. Bariatric surgery seems effective but is costly and not without risk [5,6]. In the face of this gaping unmet medical need each new potential anti-obesity drug is announced in the media with great fanfare as the next miracle pill although these have rarely been translated into effective anti-obesity therapies.

Effective anti-obesity drug therapies would either restrict energy intake or increase energy expenditure by acting either centrally or peripherally. The US Food and Drug Administration (FDA) and the European Medicines Agency (EMA) have set guidelines for what constitutes a minimally effective anti-obesity therapy. In brief the FDA requires a drug to produce a ≥5% placebo-subtracted reduction from baseline body weight due to loss of body fat after one year of treatment and the EMA requires a ≥10% body weight reduction that also must be ≥5% greater than obtained in the placebo group [7,8]. Additional secondary endpoints indicating improvements in cardiovascular and metabolic indices are also expected [7,8]. In addition, anti-obesity drugs must be relatively risk-free since they are administered chronically and both the FDA and EMA have emphasized the importance for the absence of psychiatric and cardiovascular adverse side effects [9,10].

## 2. Anti-Obesity Drug Targets in the 1990s

During the 1990s some of the more promising areas for the pharmacotherapy of obesity included central and/or peripheral neurochemical mechanisms involving serotonin (5-hydroxytryptamine, 5-HT), catecholamines and certain neuropeptides. Serotonergic and catecholaminergic mechanisms were targeted by the effective combination therapy, Fen-Phen, composed of the 5-HT releasers fenfluramine or dexfenfluramine combined with phentermine, a noradrenergic releaser [11]. The combination therapy was adopted because the drugs acted on separate monoaminergic systems and therefore might synergize, allowing for reduced dosing and, consequently, fewer side effects [12]. These expectations were met and the effective dose of each of the combined drugs was lower than either of the drugs given alone [12]. Unfortunately, even the lower dose of fenfluramine was associated with the serious side effect of cardiac vulvulopathy and this therapy was removed from the market [13]. 

Another target of promise was the peripheral β-3 adrenergic receptor. This receptor was targeted using selective agonists to increase energy expenditure through the activation of brown fat but these compounds lacked efficacy and the less selective compounds produced adverse side effects in humans [14,15,16].

At the preclinical level, a variety of neuropeptides were studied for effects on feeding with the most encouraging of these being neuropeptide-Y (NPY) which was shown to be expressed in the hypothalamus, a brain region known to control appetite. In addition, hypothalamic NPY stimulated feeding and its expression was shown to be responsive to hormonal and metabolic state indicated by induction with fasting and streptozotocin diabetes and suppression by insulin [17]. Drug discovery efforts in search of selective NPY antagonists were launched although the abundance of NPY in the brain suggested its involvement in multiple brain functions and therefore the potential for adverse side effects of NPY-based drugs.

This scarcity of potential pharmacotherapies to treat obesity was suddenly reversed with the discovery of leptin and leptin-regulated networks within the CNS. These discoveries produced a rich menu of potential anti-obesity targets (Table 1) and were pursued by all major pharmaceutical companies during the past decade.

**Table 1:** Anti-obesity Drugs.

Drugs available in 2000	New anti-obesity drug targets since the discovery of leptin	Drugs available in 2010
Orlistat	Leptin	Orlistat
Sibutramine	Leptin receptors	
	MC4 receptor	
	NPY receptors	
	PYY receptor	
	5-HT2c receptor	
	AgRP binding	
	MCH	
	Orexins	
	Incretins	
	Ghrelin	
	Endocannabinoid receptors	

## 3. The Discovery of Leptin and the Hypothalamic Circuit for Energy Homeostasis

### 3.1. Mouse Genetics Lead the Way

The discovery of leptin and its receptor in the brain [18,19] in conjunction with discoveries of the melanocortinergic pathways in the hypothalamus [20] established, for the first time, a defined hypothalamic neural circuit of feeding and satiety neurons regulated by metabolic state through the actions of hormones and nutrients [21,22]. These discoveries were made directly from monogenic mouse models. Leptin was discovered through reverse genetics and the leptin receptor by functional cloning in the pursuit of the mutations responsible for the obese, hyperphagic phenotypes of the *ob/ob* and *db/db* mouse mutants, respectively. The cellular and molecular components of the melanocortinergic pathways were discovered through studies aimed at understanding the coat color and obese, hyperphagic phenotype of the mouse *A^y^* mutant. The basic circuit, composed of three distinct neuronal subtypes, elevated analysis of energy homeostasic control by the hypothalamus from the gross anatomical level of hypothalamic areas (e.g. lateral, ventromedial, *etc.*) to defined, interacting cell phenotypes responding to defined molecular inputs with defined molecular outputs.

In both mouse and man, monogenic models of obesity, in which a spontaneous mutation in a single gene results in obesity, have been described [23,24]. All of the involved genes are either expressed in the brain or the brain serves as the major site of action of the gene products and virtually all of the genes are known to function within the defined hypothalamic neural network composed of identified interacting neuronal subtypes. As discussed below, a main function of this neural network involves the synaptic regulation of the central melanocortin receptors (MCRs) by the hormonally controlled production and release of endogenous ligands that activate or inhibit MCR activity [21,25]. This hypothalamic circuitry has been validated in rodents by genetic manipulations, anatomical studies and a large set of physiological and pharmacological studies that all strongly support the notion of a core neural network composed of hypothalamic neurons controlling body weight through the regulation of central melanocortinergic systems.

### 3.2. The Melanocortinergic System

Appreciation for the melanocortinergic regulation of body weight began with the discovery that the yellow, hyperphagic and obese mouse phenotype carrying the agouti lethal yellow (*A^y^*) mutation was due to ectopic expression of Agouti protein. Normally confined to the skin as a paracrine competitive antagonist at the melanocyte melanocortin-1 receptor (MC1R), Agouti protein is ubiquitously expressed in *A^y^* mice [26] suggesting that its ectopic expression might disrupt signaling at a MC1R-like receptor in a tissue involved in the regulation of body weight. This led to a search for MC1R-like receptors in the brain by reverse transcriptase-polymerase chain reaction (RT-PCR) using degenerate primers which led to the cloning of the brain-specific MC4R and MC3R G-protein coupled receptors (GPCRs) [27,28]. Heterologous expression of MC4R showed that it activates the heterotrimeric GTP-binding protein that stimulates adenylyl cyclase (Gs) to promote intracellular cAMP formation [27]. The endogenous MC4R agonist ligand was shown to be the pro-opiomelanocortin (POMC) product, a-melanocyte stimulating hormone (MSH) and Agouti protein proved to be a potent competitive MC4R antagonist [29]. The above results suggested that ectopic expression of the antagonist Agouti protein in Ay mice might produce hyperphagia and obesity if MC4R was involved in the central control of energy homeostasis. 

The role of MC4R in the central regulation of body weight in mice was demonstrated by the MC4R-knockout mice, which displayed an obesity phenotype identical to that of *A^y^* mice [30]. Importantly, MC4R heterozygotes displayed an intermediate obesity phenotype [30]. Overexpression of Agouti protein also reproduced the *A^y^* phenotype and data mining for Agouti protein homologs revealed an agouti-related peptide (AgRP) uniquely expressed in the brain, which, like Agouti protein, was shown to be a highly potent MC4R antagonist [31,32]. Also, like Agouti protein, overexpression of AgRP in transgenic mice reproduced the *A^y^* obesity profile [31]. In addition, syndecan-3, a cell-surface heparin sulfate proteoglycan expressed in the hypothalamus and regulated by metabolic state has been suggested to function as a co-receptor for AgRP and to facilitate AgRP binding to MC4R [33] although this has been recently questioned by additional data [34].

**Figure 1:**
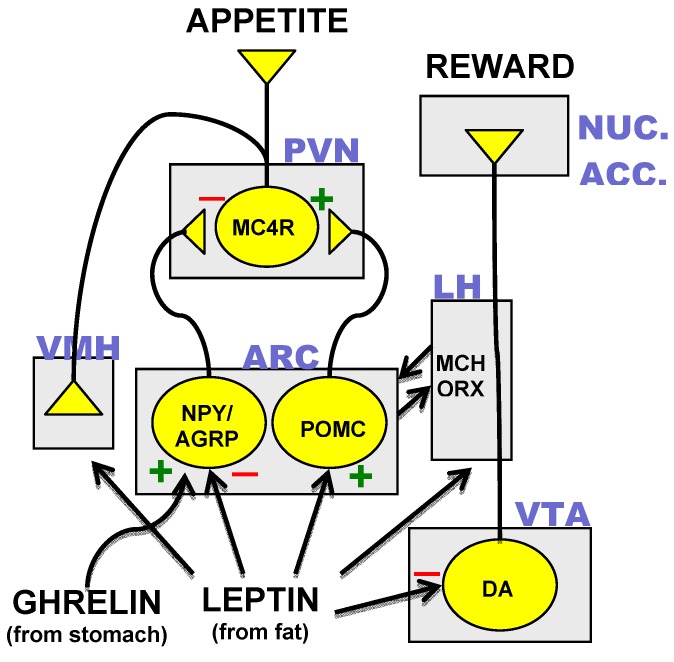
The core hypothalamic energy homeostatic circuit and the dopamine reward pathway. Neuronal subtypes expressing neuropeptide-Y and agouti related peptide (NPY/AGRP), proopiomelanocortin (POMC), the melanocortin-4 receptor (MC4R) and dopamine (DA) are in yellow. Rectangles indicate various anatomical loci: ARC = arcuate nucleus, PVN = paraventricular nucleus, VMH = ventromedial hypothalamus, LH = lateral hypothalamus, VTA = ventral tegmental area, NUC. ACC. = nucleus accumbens. Other abbreviations: MCH = melanocyte stimulating hormone. ORX = orexins. (+) = excitatory input. (−) = inhibitory input.

Identification of the neurons, which synthesized MC4R and its agonist and antagonist peptide ligands resulted in the mapping of a hypothalamic neural circuit composed of three distinct neuronal subtypes. Transcripts for AgRP were found to be co-localized with the orexigenic neuropeptide-Y (NPY) in neurons of the arcuate nucleus of the hypothalamus [35]. POMC-expressing neurons were found in close proximity to the AgRP/NPY neurons and both of these neuronal subtypes were found to project rostrally to the hypothalamic paraventricular nucleus (PVN), which contains MC4R-expressing cells [36]. Disruption of the POMC gene results in an obesity phenotype in mouse and man [37,38]. Appreciation of the neuroanatomical relationships of AgRP/NPY-, POMC- and MC4R-expressing neurons coupled with results from transgenic mouse studies [39] suggest a core hypothalamic circuit regulating energy homeostasis in which neuropeptides made in the arcuate nucleus determine MC4R activity through the interplay of MC4R agonist or antagonist neuropeptide ligands released at MC4R-expressing PVN neurons (Figure 1).

### 3.3. Hormonal Inputs to the Hypothalamic Circuit

The role of the melanocortinergic hypothalamic circuit in body weight regulation is further supported by the strong control over neuropeptide expression and neuronal activity of the arcuate neurons by metabolic hormones [20,21]. Both the AgRP/NPY and POMC arcuate neurons have been shown to express receptors for the hormone leptin, which is synthesized and secreted from fat cells and circulates in the blood to activate leptin sensitive tissues [40,41]. In the arcuate nucleus, leptin suppresses AgRP/NPY expression and inhibits firing of AgRP/NPY neurons while stimulating POMC expression and the firing of POMC neurons [42]. This combination of actions would be expected to promote activation of MC4R which appears to be a major mechanism for leptin’s anorectic effects as leptin suppression of feeding is compromised in MC4R knockout mice [43]. Conversely, mice devoid of leptin (*ob/ob*) or without a functioning leptin receptor (*db/db*) exhibit increased expression of AgRP and NPY and decreased expression of POMC. Under these conditions, the consequent reduction in MC4R activity likely promotes the hyperphagia and massive obesity of these mice although leptin also acts at other sites in the brain to suppress feeding [44]. In addition to its effects on hypothalamic neurons, leptin might also reduce the pleasure of eating by direct action on neurons of the dopamine reward pathways in the ventral tegmental area [45] (Figure 1).

Another important metabolic hormone with differential actions on the AgRP/NPY and POMC arcuate neurons is ghrelin, which is secreted from the stomach during fasting to stimulate hunger [46]. Ghrelin receptors on AgRP/NPY neurons mediate the stimulatory action of the hormone on the firing of AgRP/NPY neurons (Figure 1) that make inhibitory synapses on arcuate POMC neurons [47]. Ghrelin-induced stimulation of AgRP/NPY neurons would result in decreased MC4R activity by increased antagonist and decreased agonist release. These actions are thought to mediate ghrelin-induced feeding because mice in which both AgRP and NPY are knocked out do not exhibit feeding in response to ghrelin [48].

Plasma glucocorticoids also increase during fasting and could potentiate hunger through rapid effects on synaptic connections of POMC and AgRP/NPY neurons. In this regard, adrenalectomy is effective in reversing the genetic obesity of *ob/ob* mice [49]. Recently, glucocorticoids have been shown to decrease inhibitory synaptic inputs to POMC neurons and to promote the formation of excitatory inputs to AgRP/NPY neurons [50]. Alternatively, or in conjunction with these effects, glucocorticoids could influence feeding through actions on neurons of the PVN [51,52]. Indeed, by virtue of the widespread expression of the glucocorticoid receptor, all areas represented in Figure 1 could potentially exhibit important regulation by glucocorticoids [53].

Appreciation of this hypothalamic neuronal circuit (Figure 1) defined by its metabolically sensitive expression and release of neuropeptides and receptors generated an extensive menu of anti-obesity drug targets of great potential for effective anti-obesity therapies. The following is a list of these targets and the current status of associated drug discovery efforts.

## 4. CNS Anti-Obesity Drug Targets Since the Discovery of Leptin

### 4.1. Leptin

The absence of the fat cell-derived hormone leptin, in both mice and humans with loss-of-function leptin mutations, signals to the brain that energy stores of fat are depleted and the organism should decrease metabolism, activity and reproductive behavior and increase energy (food) intake [54]. As a result, animals without leptin become grossly obese. Initial hope that obese patients suffered from hypoleptinemia and that obesity might be treated by leptin replacement therapy was quickly discarded when obese rodents and humans were found to be hyperleptinemic. The concept of “leptin resistance” was developed in light of experiments showing that rodents fed a high fat diet (HFD) for one week showed reduced behavioral responsiveness (*i.e.* reduced suppression of feeding) and reduced molecular effects in the hypothalamus (*i.e.* a reduction in the phosphorylation of a key signaling molecule of leptin, signal transducer and activator of transcription-3, Stat3) in response to leptin administration [55]. In this obesity model, high fat feeding promoted leptin resistance either through desensitization due to hyperleptinemia from HFD-induced expansion of adipose tissue or by direct action of overnutrition on leptin-sensitive cells. Once leptin resistance set in, energy homeostasis would be further compromised. In this view, leptin resistance, marked by hyperleptinemia and the inability of leptin to act, would be analogous to insulin resistance and hyperinsulinemia of Type II diabetes. As such, the potential mechanisms of leptin resistance have been a major target of anti-obesity research much as overcoming insulin resistance has been the focus of research to reverse Type II diabetes. 

One view of leptin resistance states that the excess nutrients of a HFD can generate an inflammatory response in hypothalamic neurons which renders them resistance to leptin [56,57]. A recent study shows a clear dissociation between HFD-induced obesity and leptin resistance. In this study, mice prevented from developing HFD-induced hyperleptinemia, did not develop HFD-induced leptin resistance but showed the normal HFD-induced obesity indicating that hyperleptinemia is required for leptin resistance [58]. Whether the hyperleptinemia then initiates a hypothalamic inflammatory response or whether the inflammation is not involved in leptin resistance remains to be determined. Nonetheless, if leptin sensitivity could be preserved in the obese, hyperleptinemic state it is possible that endogenous or exogenous leptin might limit or reverse the obesity. In this regard, the minimal weight loss effects of a methionyl recombinant human leptin, metreleptin, has been shown to be potentiated by pramlintide (Amylin Pharmaceuticals, Inc., San Diego, CA, USA), an analog of the β-cell hormone, amylin, that has been shown to reverse HFD-induced leptin resistance in mice [59]. Importantly, pramlintide/metreleptin treatment has been shown to effectively reduce weight in humans [60] and Amylin Pharmaceuticals, in collaboration with Takeda Pharmaceutical Co., Ltd (Osaka, Japan), are currently planning phase III clinical trials for the anti-obesity combination therapy [61]. In addition, sensitivity to leptin is heightened in mice treated with the PTP(protein tyrosine phosphatase)-1B inhibitor trodusquemine (MSI-1436, Genaera Corp., Plymouth Meeting, PA, USA) and this compound is presently in phase II clinical trials [62]. The phosphatase is thought to remove the phosphates from tyrosine activation sites of the leptin receptor and the PTP-1B inhibitor would therefore potentiate leptin action. To date, however, the promise of leptin as an anti-obesity therapy remains to be realized.

### 4.2. NPY and AgRP

Central administration of NPY and AgRP induce vigorous feeding responses in rodents and the modulation of their actions by drugs might be expected to affect appetite. Although the deletion of NPY and AgRP genes did not result in robust metabolic phenotypes, subtle effects were noted in obese and ageing mice [63,64,65] Each of these neuropeptides presents unique drug discovery challenges. 

NPY is widely expressed in the brain and is therefore involved in diverse brain systems subserving a variety of functions [66]. NPY effects are mediated by at least five different G-protein coupled receptor (GPCR) subtypes with Y-1 and Y-5 subtypes identified as mediating the orexigenic effects of NPY [67,68,69]. Conversely, activation of the presynapticY-2 and Y-4 receptors by selective agonists has been shown to inhibit appetite, presumably by inhibiting release of orexigenic factors although Y-2 antagonism has been shown to act in fat tissue to block fat accumulation [70]. Y-2 and Y-4 receptors are also thought to mediate the anorexic effects of the gut hormone peptide-YY (PYY) and pancreatic polypeptide (PP), respectively. After disappointing clinical trials, at least one company (Merck & Co., Inc., Whitehouse Station, NJ, USA) has abandoned its Y-5 antagonist (MK-0557) as an antiobesity therapy [71] but Shionogi (Osaka, Japan) is continuing to test its Y-5 antagonist, velneperit, for use in combination with an intestinal lipase inhibitor [72,73].

AgRP presents a different challenge since it operates as an endogenous antagonist, competitively blocking the binding of the anorexigenic neuropeptide, α-MSH, to the MC4R. An effective anti-obesity AgRP antagonist would have to prevent AgRP binding without blocking the binding of α-MSH. This is conceivable given the apparent accessory binding to a syndecan co-receptor required by AgRP for binding to MC4R [33] although a recent report questions whether this binding occurs when AgRP is fully processed *in vivo* [34] Selective antagonism of AgRP with a co-receptor could reduce AgRP binding to MC4R without affecting α-MSH binding. Transtech Pharma, Inc. (High Point, NC, USA) has reported the development of a selective AgRP antagonist, TTP-435, that has recently completed Phase II clinical trials [74].

### 4.3. MC4R

Deletion or haploinsufficiency of MC4R results in obesity in mouse and man [75,76,77,78]. Indeed, loss-of-function mutations of MC4R in humans can result in obesity that is resistant to bariatric surgery [79]. Pharmacological administration of MC4R agonist and antagonist ligands have been shown to, respectively, suppress and stimulate feeding behavior in rodents. Acute activation of MC4R by central administration of α-MSH or the synthetic agonist MTII have been shown to suppress feeding and blockade of MC4R by AgRP (see above) or the synthetic antagonist SHU9119 have been shown to promote feeding [36,80,81]. These ligands do not discriminate between MC4R and MC3R, and therefore some of the effects could be mediated via MC3R although, based on gene knockout and association studies, it would appear that MC4R is the major MCR controlling appetite [30,82,83].

Up to 6% of patients with early onset obesity have been reported to carry loss-of-function mutations of MC4R [75,77,84,85]. Pharmacological approaches to rescue certain genetically defective MC4R receptors have involved the synthesis of novel agonists or compounds that serve as endoplasmic reticular (ER) chaperones or modulate ER protein degradation [86,87,88,89]. The strong association of MC4R mutations with obesity across a wide variety of human populations genetically validates the critical role of this receptor in human energy homeostasis. Indeed, the association of haploinsufficiency of MC4R with obesity suggests that the receptor is a bottleneck for the flow of metabolic information within the energy homeostatic system. As such, much effort has been expended in the search for effective MC4R agonists as anti-obesity therapies.

There are at least three important challenges that arise in the search for effective MC4R anti-obesity drugs. First, the search is for agonists, which must bind and then activate the receptor unlike antagonists, where only receptor binding is required. Also, given the obesity phenotype associated with loss-of-function of a single MC4R allele, it would appear that full agonists might be required and that partial agonists with low enough intrinsic activity could actually oppose the desired effects. Second, as the name implies, MC4R is a member of the melanocortinergic subgroup of GPCRs which consists of five family members with diverse physiological functions and therefore raising the requirement for subtype selectivity [75]. Third, MC4R is widely expressed within the central nervous system and is, itself, involved in a variety of physiological functions, raising the specter of adverse side effects even if the agonist and selectivity requirements for an anti-obesity MC4R-targeted activator were met.

Recently, the overall drug discovery approach for anti-obesity MC4R agonists has been complicated by studies linking MC4R activation with therapeutic effects for erectile dysfunction, sexual libido and inflammation on the one hand and adverse elevations in blood pressure [90,91,92,93,94]. Although effects on sexual function and inflammation could prove problematic, it is clear that the major threat to the use of MC4R anti-obesity agonists is posed by their likely hypertensive effects. It remains to be seen whether this serious concern can be obviated. If MC4R control over energy homeostasis proved more sensitive to agonist activation, body weight and cardiovascular effects might be dissociable by calibration of agonist dosing as recently tested on sexual dysfunction and blood pressure effects of the melanocortin agonist, bremelanotide [95]. If sites for MC4R energy homeostatic control are anatomically distinct from those affecting cardiovascular functions, differential modulation of MC4R activation could be attempted through targeting of accessory proteins such as mahogunin and β-defensins if expression of these proteins were site-specific [96,97,98]. Alternatively, the different sites could be mined for signaling molecules downstream of MC4R by transcriptome analysis [99] and targeted if expressed in a site-specific manner.

### 4.4. 5-HT

Although Fen-Phen was removed from the market in light of its adverse side effects, the combined action of fenfluramine and phentermine, to release serotonin and catecholamines, respectively, proved to be an effective weight loss treatment. It is not surprising, therefore, that sibutramine, a non-selective 5-HT and noradrenergic re-uptake inhibitor proved to have some weight-reducing efficacy [100]. However, after nine years of sales, this drug has been removed from the European market due to elevated risk for nonfatal stroke and myocardial infarction and has received a contraindication for use in patients with cardiovascular disease from the US Food and Drug Administration [101,102,103] and this has led to its withdrawal from the US market by Abbott Laboratories [104]. Nonetheless, serotinergic mechanisms continue to be targeted in the search for anti-obesity therapies. Indeed, tesofensine, a 5-HT-norepinephrine/dopamine re-uptake inhibitor (NeuroSearch A/S, Ballerup, DK), has shown efficacy in early clinical studies [105] and phase III clinical trials are scheduled to begin in late 2010.

Data from a variety of transgenic mouse experiments show that the 5-HT receptor subtypes, 5-HT1b and 5-HT2c, control the activity of key neurons within the energy homeostasis circuit of the hypothalamus. Activation of these receptors, like activation of the leptin receptor on these cells, either stimulate the release of the anorectic neuropeptide, α-MSH or inhibit activity in orexigenic AgRP/NPYneurons [106,107]. The targeting of specific 5-HT receptor subtypes that may not subserve cardiovascular functions is likely to avoid cardiovascular liability. In this regard, a new potential anti-obesity 5-HT2c agonist, lorcaserin (Arena Pharmaceuticals, Inc., San Diego, CA, USA) has recently been shown to have modest efficacy (≥5 kg weight loss over placebo after one year) in obese or overweight patients with no cardiovascular involvement reported [108]. However, due to concerns over possible cancer risks, an FDA advisory panel recently recommended against advancing locarserin to the market [109].

### 4.5. Melanin Concentrating Hormone (MCH) and Orexins

Neurons expressing either MCH or orexins are abundant in several hypothalamic sites including the lateral hypothalamus and exhibit reciprocal connectivity with POMC and AgRP/NPY neurons in the arcuate nucleus (Figure 1) although the specific impact of these connections on neuropeptide synthesis and release remains unresolved [110,111]. Administration of MCH or orexins to rodents stimulates feeding and it is thought that blockade of specific receptor subtypes for these neuropeptides could serve to inhibit appetite. In mice, administration of an MCH-1 receptor antagonist decreased body weight through decreased feeding and increased energy output [112]. 

AMRI (Albany, NY) has recently begun a Phase I clinical trial for ALB-127158(a), an MCH-1 receptor antagonist, for anti-obesity therapy [113]. Actelion Pharmaceuticals, Ltd (San Francisco, CA, USA), in collaboration with GlaxoSmithKline plc (Brentford, UK) has recently announced completion of short term Phase III clinical trials for almorexant, a non-selective orexin-receptor antagonist, for the treatment of sleep disorders [114] and Merck & Co., Inc. has also begun clinical trials for its orexin receptor antagonist, MK-4305, also targeting sleep disorders [115,116] yet it would seem that these compounds could have anti-appetite actions as well [117]. In addition, MCH and the orexins also promote mechanisms of drug addiction [118,119,120,121] with predicted anti-addiction properties for antagonists although blockade of reward pathways could entail liability for mood disorders and depression (see Endocannabinoids below).

### 4.6. Ghrelin

The orexigenic AgRP/NPY neurons are directly activated by ghrelin, a hormone synthesized and released from the stomach in response to fasting [122]. Ghrelin stimulates feeding in rodents and it has been shown to induce expression of c-fos (a marker of neuronal activity) in AgRP/NPY neurons [123,124]. Receptors for ghrelin are present in AgRP/NPY neurons and electrophysiological experiments have shown direct activation of AgRP/NPY neurons by this hormone [125,126]. As mentioned above, rodents without AgRP and NPY do not increase feeding in response to ghrelin [48]. The fact that ghrelin levels correlate with hunger, rising during fasting and just prior to a meal [123,127], suggest that blockade of ghrelin action could be an effective appetite suppressant [128]. This view is strengthened by evidence of a potential link between the success of bariatric surgery to decrease body weight and lowered postoperative ghrelin levels [129].

Although ghrelin interacts with other physiological systems in addition to energy homeostasis [128], it is possible that a ghrelin antagonist could serve as an effective anti-obesity therapy without adverse side effects. The ghrelin knock-out mouse has no obvious phenotype with discrepant reports on whether loss of the ghrelin gene produces resistance to a HFD [130,131] although a more recent report indicates a generally improved metabolic profile of high-fat fed ghrelin knockout mice [132]. Elixir Pharmaceuticals, Inc. (Cambridge, MA, USA) has developed a small molecule ghrelin receptor antagonist currently in early stage human trials. Another approach would be to target the enzyme, ghrelin *O*-acyltransferase (GOAT), responsible for the post-translational activation of ghrelin. Biological activity of ghrelin is entirely dependent upon its acylation with an eight-carbon fatty acid and this reaction is uniquely catalyzed by GOAT [133]. An enzyme inhibitor directed at GOAT would decrease the levels of biologically active ghrelin.

### 4.7. Incretins

The incretin hormones, glucagon-like peptide-1 (GLP-1) and glucose dependent insulinotropic polypeptide (GIP), are posttranslational proteolytic products of the proglucagon gene and are released from the intestinal mucosa in response to nutrient intake [133]. GLP-1 activation of its GPCR receptors in a variety of tissues results in delayed gastric empyting, increased glucose-stimulated insulin release from pancreatic β-cells, decreased release of glucagon from pancreatic α-cells and suppression of appetite [134]. The satiety induced by peripheral administration of GLP-1 or GLP-1 analogues might be mediated through decreased gastric emptying or other peripheral mechanisms or by central action on the hypothalamic circuit for energy homeostasis or brainstem [135,136,137,138]. 

GLP-1 is rapidly inactivated in the blood by dipeptidyl peptidase IV (DPP-4) and this challenge has been addressed by the development of GLP-1 analogues that are DPP-4 resistant, such as exenatide (Amylin Pharmaceuticals, San Diego, CA and Eli Lilly & Co., Indianapolis, IN, USA) and liraglutide (Novo Nordisk, Inc., Princeton, NJ, USA) and by the development of DPP-4 inhibitors such as sitagliptin (Merck & Co., Inc) and saxagliptin (Bristol Myers Squibb, New York, NY, USA) to elevate endogenous level incretin levels. Only the GLP-1 analogues have been shown to reduce weight while DPP-4 inhibitors are weight neutral and GIP is either weight neutral or promotes adiposity through lipogenic action on adipocytes [139,140,141,142].

Both GLP-1 analogues are approved as anti-Type2 diabetes mellitus (T2DM) drugs and are typically prescribed in conjunction with other anti-T2DM medications. They are not used as weight reduction medications per se although the weight-reducing effects of these agents likely play an important role in their overall anti-diabetic profile [139,140]. A post-operative rise in GLP-1 has been attributed to the efficacy of bariatric surgery to produce weight loss and improve glucose control in morbidly obese patients [143,144,145]. Another proglucagon gene processing product from intestinal mucosa cells is oxyntomodulin which has also been shown to inhibit appetite and may also be involved in positive outcomes of bariatric surgery [146,147]. A oxyntomodulin analogue, TKS1225 (Thiakis Limited, London, UK) was advanced to Phase I clinical trials and acquired by Wyeth (Madison, NJ, USA) which is now part of Pfizer, Inc. (New York, NY, USA). Other gut peptides such as PYY and cholecystokinin remain to be translated into effective anti-obesity therapies.

### 4.8. Endocannabinoids

Efforts to understand the mechanisms of action of the exogenous cannabinoid, Δ^9^-tetrahydrocannbinoid (Δ^9^-THC) resulted in the elaboration of the endocannabinoid (EC) system composed of EC ligands, synthetic and catabolic enzymes and EC ligand receptors [148,149]. One effect of Δ^9^-THC is to stimulate feeding and the EC system has been shown to interact with the hypothalamic circuit for energy homeostasis with a profile suggesting a direct relationship between energy intake and EC system activation [150,151,152,153,154]. The CB1 receptor subtype was shown to mediate effects on feeding [155] leading to the development of CB1 receptor antagonists to inhibit feeding. Further, recent work, in high fat-fed mice, has shown that chronic treatment with the CB1 receptor antagonist, rimonabant (Sanofi-Aventis, Paris, France), produces weight loss and other metabolic improvements that are independent of decreased intake [156,157].

Thus, selective CB1 antagonists were highly promising anti-obesity therapies. Such expectations were validated as rimonabant was shown to reduce body weight and improve metabolic parameters in a number of clinical trials leading to the successful marketing of the drug in Europe as an anti-obesity therapy in 2006 [158]. However, in 2007, the FDA failed to approve rimonabant over concerns of association of the drug with psychiatric adverse events and suicidality [159]. This decision led to increased monitoring of the drug in Europe, restricting indications to exclude patients with a history of psychiatric disorders, and its eventual removal from the European market in 2008 [158,160]. 

The involvement of central CB1 receptors in the functioning of central motivational pathways most likely underlies the adverse psychiatric effects seen with rimonabant [161,162,163,164]. This result does not necessarily rule out the CB1 receptor as an anti-obesity drug target; the receptor antagonism achieved by rimonabant, an inverse agonist, could be overkill and a neutral antagonist or partial agonist, which would not silence constitutively active CB1 receptors, might reduce weight without adverse side effects [158]. Alternatively, it is possible that blocking peripheral CB1 receptors, thereby avoiding direct central action, could have anti-obesity benefits in humans since peripherally acting CB1 antagonists have recently been shown to reverse several unfavorable metabolic indices induced by HFD in mice [165,166]. Nonetheless, the failure of rimonabant serves as a cautionary lesson that centrally acting drugs run the risk of producing adverse psychiatric side effects especially if their mechanism of action is inextricably linked to the functioning of midbrain dopamine reward pathways [167,168,169]. 

### 4.9. New Combination Therapies

In light of the above considerations, Qnexa, the combination anti-obesity therapy consisting of phentermine and the anti-convulsant topiramate, put forth by Vivus, Inc. (Mountain View, CA, USA) was recently rejected for marketing by an FDA advisory panel [170]. It remains to be seen what the future holds for a new anti-obesity combination therapy of naltrexone, an opiate antagonist, and bupropion, a catecholamine re-uptake inhibitor and nicotinic antagonist, known as Contrave (Orexigen Therapeutics, Inc., La Jolla, CA, USA) which targets both hypothalamic POMC neurons and the midbrain dopamine neuron reward pathway. [171,172]. Empatic, also from Orexigen Therapeutics, a combination anti-obesity therapy of bupropion and the anti-convulsant, zonisamide, has shown promise in phase II clinical trials and phase III trials are being planned [173,174]. The rationale for combination therapy is based on the likelihood that mechanisms of energy homeostasis might defeat a single monotherapy whereas affecting multiple targets would stand a better chance at blocking homeostatic responses by reducing the activity in compensatory pathways and/or through synergistic effects on separate but ultimately converging pathways [12].

## 5. Peripheral Anti-Obesity Drug Targets Since the Discovery of Leptin

CNS anti-obesity drug targets present major hurdles to drug development programs. To gain access to central neurons, a compound must navigate the blood brain barrier and then act selectively amidst a complex and interconnected target-rich tissue to produce its effect without adverse side effects [175,176]. Peripheral targets are far more likely to be successfully modulated by drugs, or, in the parlance of drug discovery, they are more “druggable”.

### 5.1. Pancreatic Lipase

Pancreatic lipase is an enzyme made in the exocrine pancreas that is secreted into the intestinal lumen to breakdown ingested triaglycerides to fatty acids and 2-monoglycerides for absorption by the gut. To date, only one peripherally-targeted drug, Orlistat, has been developed as an anti-obesity therapy although other compounds, such as the incretins and endocannabinoid antagonists, could produce at least part of their anti-obesity effects at peripheral sites. Orlistat (Orlistat, F. Hoffmann-LaRoche, Basel, Switzerland and Alli, GlaxoSmithKline plc, Brentford, UK) is a lipase inhibitor which prevents the breakdown of triglycerides in the intestine and therefore decreases fat absorption. Orlistat is the ultimate peripherally-acting compound in that the drug, itself, is minimally absorbed, having its action in the lumen of the gut. However, the drug is, at best, only modestly effective and its use is accompanied by unpleasant gastrointestinal side effects which limit its long-term use [177] and reduce patient compliance [178]. The FDA has recently revised the label for Orlistat to include reports of rare cases of severe liver injury in patients on this medication [179]. A related lipase inhibitor, Cetilistat (Norgine BV, Amsterdam, The Netherlands, in collaboration with Takeda Pharmaceutical Co., Ltd., Osaka, Japan), that may be better tolerated, is currently in phase III clinical trials [180].

### 5.2. 11β-Hydroxysteroid Type 1

Despite strong evidence from rodent models for the permissive role of glucocorticoids in a variety of obesity models, there is little evidence for elevated plasma glucocorticoid levels in common forms of human obesity [181]. These observations have led to interest in 11β-hydroxysteroid type 1, the enzyme required for the reactivation of cortisol from cortisone. Increased reactivation of cortisol by the enzyme could promote glucocorticoid action in cells without elevated plasma levels of the hormone. Overexpression of the enzyme specifically in mouse adipose tissue resulted in increased corticosterone levels in fat tissue without elevations in plasma glucocorticoids while increasing visceral fat mass and generating other obesity-associated metabolic complications [182]. As such, there is great interest in the development of 11β-hydroxysteroid type 1 inhibitors and a large array of small molecule inhibitors have been generated by a number of pharmaceutical companies [183]. Early-stage clinical trials are underway for several of these compounds.

### 5.3. Human Brown Adipose Tissue (BAT)

Anti-obesity therapies are typically aimed at reducing energy intake although some might also have the additional effect of increasing expenditure. The targeting of BAT represents an effort to uniquely target energy expenditure by driving the metabolic activity of this tissue; an approach that has been shown to have powerful anti-obesity effects in rodents [184]. Drug discovery efforts in the 1990s included the targeting of BAT via β-3 adrenergic receptor activation since activation of this receptor in rodents promotes a powerful anti-obesity effect through the induction of lipolysis in white adipose tissue and the oxidation of free fatty acids in BAT [16]. However, selective β-3 adrenergic receptor agonists did not prove efficacious in humans and this failure was most likely due to the paucity of this receptor subtype in human adipose tissue relative to that found in rodents [185,186]. Much has been made of the recent validation of the presence of BAT in humans [187,188,189,190,191] but it still remains to be demonstrated that activation of this tissue in adult humans would produce clinically beneficial effects on body weight. Investigation into the molecular determinants of the BAT phenotype could provide clues for the potential expansion of BAT mass for future anti-obesity therapies [184].

### 5.4. Targets That Could Limit Consequences of Obesity

Even if efficacious anti-obesity drug therapies are developed there will likely be many obese patients that will require treatment for their existing obesity. Therefore natural or synthetic products will be necessary to combat the consequences of obesity, such as inflammation and insulin resistance to limit obesity-induced pathologies such as Type 2 diabetes and dyslipidemias. Among these targets are adiponectin and its receptors [192,193], the metabolic regulator AMP-activated protein kinase (AMPK) [194,195,196], PPARs [197], sirtuin-1 [198] and GPCR 120 [199]. Although drugs for these targets would most likely not be indicated for obesity prevention *per se*, and, in some cases such as peroxisome-profilerator-activated receptors γ (PPARγ) agonists, could even increase adiposity [200,201], they could be used to maintain insulin sensitivity and limit inflammation in the obese state. On the other hand, compounds such as PPARα and PPARδ agonists could actively decrease fat stores through induction of fatty acid oxidative pathways [196,202]. To date, few drugs for these targets have been adopted for long-term effective treatment of obese patients and recently, use of the PPARγ agonist, rosiglitazone, has been suspended in Europe and sharply restricted in the US [203,204].

## 6. Concluding Remarks

At least 10 years is typically required to go from target discovery/validation to regulatory agency approval of a new drug therapy. From this perspective, it is not surprising that many new drug discovery programs for anti-obesity targets discovered since the discovery of leptin have not led to new drug therapies. Far more troubling is the fact that several new chemical entities have successfully navigated through to various stages of the process, with some actually being approved, only to be dropped or restricted in light of new evidence regarding safety or lack of efficacy. Among these are sibutramine, MC4R agonists, lorcaserin, rimonabant and Qnexa. Moreover, several of the new compounds nearing evaluation by regulatory agencies are similar to those already rejected. The GLP-1 agonists, which appear to have both central and peripheral appetite reducing effects, are primarily Type 2 diabetes drugs given their powerful promotion of glucose-stimulated insulin release and may not be prescribed as anti-obesity drugs *per se*. 

Thus, at 2000, the choice of approved long-term anti-obesity drug therapies was between Orlistat and sibutramine (Table 1). Now, a decade later at 2010, there is only Orlistat (Table 1). At present, the obesity epidemic grows unabated generating more cases of Type 2 diabetes, dyslipidemias, cardiovascular disease and other obesity-related disorders. Diet and exercise remain effective but have poor compliance. Expensive bariatric surgery is effective for cases of morbid obesity but is not an option for 300 million obese patients. It is hoped that the next decade will produce effective anti-obesity drug therapies to at least partially fill this expanding unmet medical need. Whether the failures of the last ten years will provide sufficient feedback for course correction, or whether the last ten years will be considered a “lost decade” for anti-obesity drug discovery remains to be seen.
